# Blunted dynamics of adenosine A_2A_ receptors is associated with increased susceptibility to *Candida albicans* infection in the elderly

**DOI:** 10.18632/oncotarget.11760

**Published:** 2016-08-31

**Authors:** Lisa Rodrigues, Isabel M. Miranda, Geanne M. Andrade, Marta Mota, Luísa Cortes, Acácio G. Rodrigues, Rodrigo A. Cunha, Teresa Gonçalves

**Affiliations:** ^1^ CNC-Centre for Neuroscience and Cell Biology, University of Coimbra, Coimbra, Portugal; ^2^ FMUC-Faculty of Medicine, University of Coimbra, Coimbra, Portugal; ^3^ Department of Microbiology, Cardiovascular Research & Development Unit, CINTESIS-Center for Health Technology and Services Research, Faculty of Medicine, University of Porto, Porto, Portugal; ^4^ Department of Physiology and Pharmacology, Federal University of Ceará, Ceará, Brazil

**Keywords:** ageing, infection, gut, Candida albicans, adenosine A_2A_ receptors, Gerotarget

## Abstract

Opportunistic gut infections and chronic inflammation, in particular due to overgrowth of *Candida albicans* present in the gut microbiota, are increasingly reported in the elder population. In aged, adult and young mice, we now compared the relative intestinal over-colonization by ingested *C. albicans* and their translocation to other organs, focusing on the role of adenosine A_2A_ receptors that are a main stop signal of inflammation. We report that elderly mice are more prone to over-colonization by *C. albicans* than adult and young mice. This fungal over-growth seems to be related with higher growth rate in intestinal lumen, independent of gut tissues invasion, but resulting in higher GI tract inflammation. We observed a particularly high colonization of the stomach, with increased rate of yeast-to-hypha transition in aged mice. We found a correlation between A_2A_ receptor density and tissue damage due to yeast infection: comparing with young and adults, aged mice have a lower gut A_2A_ receptor density and *C. albicans* infection failed to increase it. In conclusion, this study shows that aged mice have a lower ability to cope with inflammation due to *C. albicans* over-colonization, associated with an inability to adaptively adjust adenosine A_2A_ receptors density.

## INTRODUCTION

Over the past decades, the number of opportunistic fungal diseases has increased, taking advantage of debilitated physiological conditions or of local or systemic immune malfunction [1, 2]. Fungal infections have a particular incidence in individuals above 65 years that also display more frequently signs of immunosenescence [3]. Furthermore, inflammation increases with age [4] and can shift the balance between commensalism and infection towards the latter [5], making aged individuals more susceptible to opportunistic infections.

*Candida albicans* is often described as commensal, being part of the human microbiota. However, when the immune status and/or the host microbiota are altered, commensalism evolves toward extensive colonization of mucosal surfaces and disease. In these cases, gut is a main gateway for *C. albicans* colonization [6]. The imbalance between pro- and anti-inflammatory signals, responsible for keeping the host-fungus equilibrium, can result in severe host damage, such as chronic inflammation, but also renders fungal cells more aggressive [7]. Adenosine, through the recruitment of adenosine receptors, in particular A_2A_ receptors (A_2A_R) is a main signal decreasing gut inflammation [8, 9]. This major controller of the reactivity of the immune-inflammatory system [10] undergoes a decreased capacity to dampen inflammation in some organs upon ageing [11], but it is unknown if A_2A_R density is modified in the gut of aged individuals.

Our objective was to understand, using a murine model, the relation between host age and susceptibility to over-colonization or infection by *C. albicans,* and the resulting inflammation of the gut. We also aimed to explore and correlate the localization and density of adenosine A_2A_R in the gastrointestinal tract with age and *C. albicans* over-colonization. The *in vivo* results indicate that ageing favors the over-colonization of the intestinal lumen and that this bolsters inflammatory signs. Moreover, aged mice display a lower A_2A_R density, which remains unchanged upon *C. albicans* infection, in contrast to young or adult mice.

## RESULTS

### *C. albicans* load in mice stools and target organs

**Figure 1:**
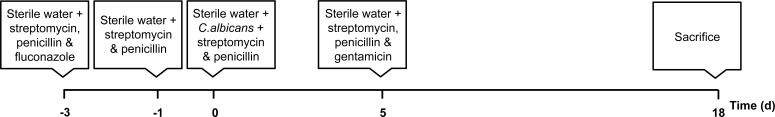
Schematic timeline of the gastrointestinal murine model of infection Young, adult and aged mice were first maintained with antibiotics in the drinking water to eliminate the gut microbiota (days −3 to 0); this allows an oral infection with *C. albicans* (days 0 to 5) to trigger an effective colonization of the GI tract (days 5-18). Mice were sacrificed at day18 for collection of tissues for analysis.

Since mice are not normally colonized by *C. albicans*, we made use of an established protocol for gastrointestinal (GI) colonization [12, 13] (Figure 1); this requires an initial elimination of the existent microbiota with antibiotics followed by infection of the mice by oral intake of a yeast suspension in the drinking water. To exclude the possibility of microbiota transference between different animals [14, 15, 16], mice were housed individually. We confirmed *C. albicans* identity in stools and tissues using a PCR analysis and correspondent ITS-5.8S sequencing (data not shown). In control animals (without *C. albicans* administration), no yeast cells were found in stools (data not shown). Using this GI infection model, none of the mice from the different groups suffered significant weight alterations (data not shown) and no mice died during the experiments, in line with previous studies [14].

**Figure 2:**
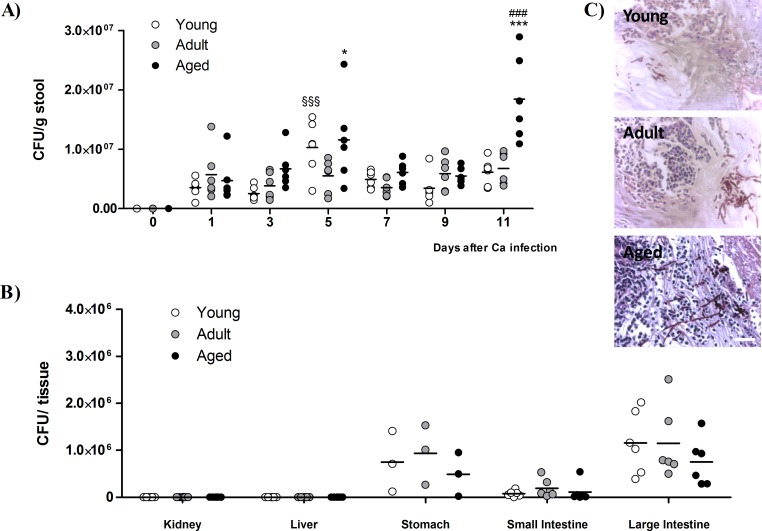
Yeast load in mice stools and target organs **A.** Stool fungal burdens of young, adult and aged mice before (day 0) and in consecutive days after *C. albicans* oral infection. Stools were collected every two days until the end of the experiment, from mice housed individually. **B.** Viable *C. albicans* counts in different tissues of young, adult and aged mice at the end of the experimental procedure (day 18). Dots represent data from individual mice and horizontal lines represent the mean of each group of mice; * *p* < 0.05, *** *p* < 0.001, aged *vs.* adult mice; ^###^ *p* < 0.001, aged *vs.* young mice and ^§§§^*p* < 0.001, adult *vs.* young mice. **C.** *C. albicans* hyphal forms were found in stomachs from young, adult and aged mice 18 days after infection with *C. albicans*. Scale bar: 5 μm.

To check GI tract colonization, the fungal load of stools was monitored every two days; as expected, at day 0, fungal cells were absent from the mice commensal flora (Figure 2A). Along the experiment, following the infection with *C. albicans*, the levels of yeasts elimination in adult mice remained constant, beginning to be detected at day 1, with values between 3×10^6^ and 7×10^6^ CFU/g of stool (Figure 2A). During the initial period of *C. albicans* infection *per os,* the stool of young and aged mice displayed similar values of fungal load, although with some point differences. In the last day of *C. albicans* administration (day 5), the number of yeast cells excreted by young and aged mice was significantly higher than in adult animals (~1×10^7^ CFU/g stool, *p* < 0.001 and *p* < 0.05, respectively, *vs*. adult mice; Figure 2A). Between days 7 and 9, the number of viable yeast cells eliminated in the stools of young and aged mice became similar to the profile found in adults until the end of the study, except that in the last time point (day 11) where aged mice had a higher number of yeast cells in the stools when compared with the other age groups (~2×10^7^ CFU/g stool, *p* < 0.001; Figure 2A).

At the end of the study, the animals were sacrificed and the fungal content was investigated in kidneys, livers, stomachs, small and large intestines. No yeast colonization was found in either kidneys or livers from any group (Figure 2B). *C. albicans* cells were found in all the gastrointestinal tract tissues, stomach, small intestine and large intestine from infected mice of the three age groups. The results for fungal content had high variability (values ranging from ~8×10^4^ to ~1×10^6^ CFU/g of tissue), and the samples taken from the large intestine of the three age groups displayed larger fungal loads than the other GI tissues (~1×10^6^ CFU/g tissue; Figure 2B). However, when comparing between the three groups, aged mice tend to present a lower colonization level in the stomach and intestine, although not statistical significant compared to adult mice (Figure 2B). The histological analysis revealed that, at day 11, the colonization of the stomach mucosa was more evident than in the intestine (small and large). Interestingly, *C. albicans* yeast-to-hyphae transition in the stomach was more exuberant in aged mice than in young and adult mice (Figure 2C).

### Inflammation processes in the GI tract

**Figure 3:**
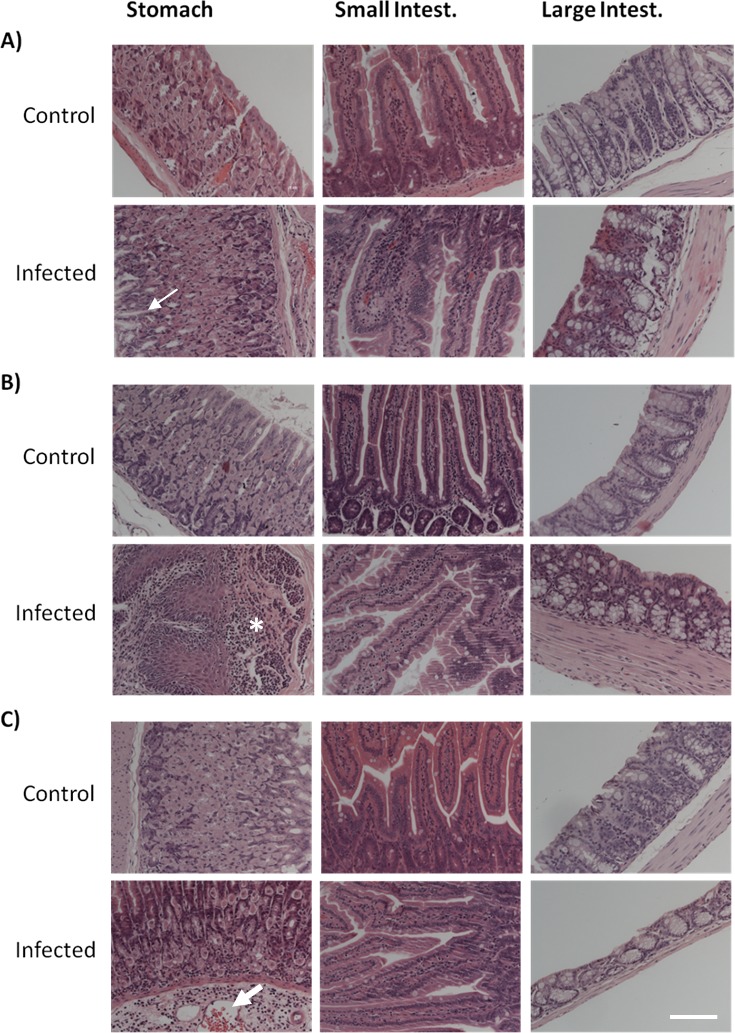
Histological analysis of haematoxylin-eosin (HE) stained tissues Stomach, small and large intestine sections from both control and *C. albicans* infected mice from **A.** aged, **B.** adult and **C.** young groups. Images from infected mice illustrate *C. albicans* infection capacity to induce severe degree of villi disruption, hemorrhagic damage and inflammatory cells infiltration on gastric mucosa, particularly evident in aged animals. Photographs point to the yeast-induced epithelial cell loss (thin arrow), inflammatory infiltrate (asterisk), and hemorrhage (large arrow). Images were taken using a Zeiss Axio Imager Z2 Microscope; scale bar: 50 μm.

**Figure 4:**
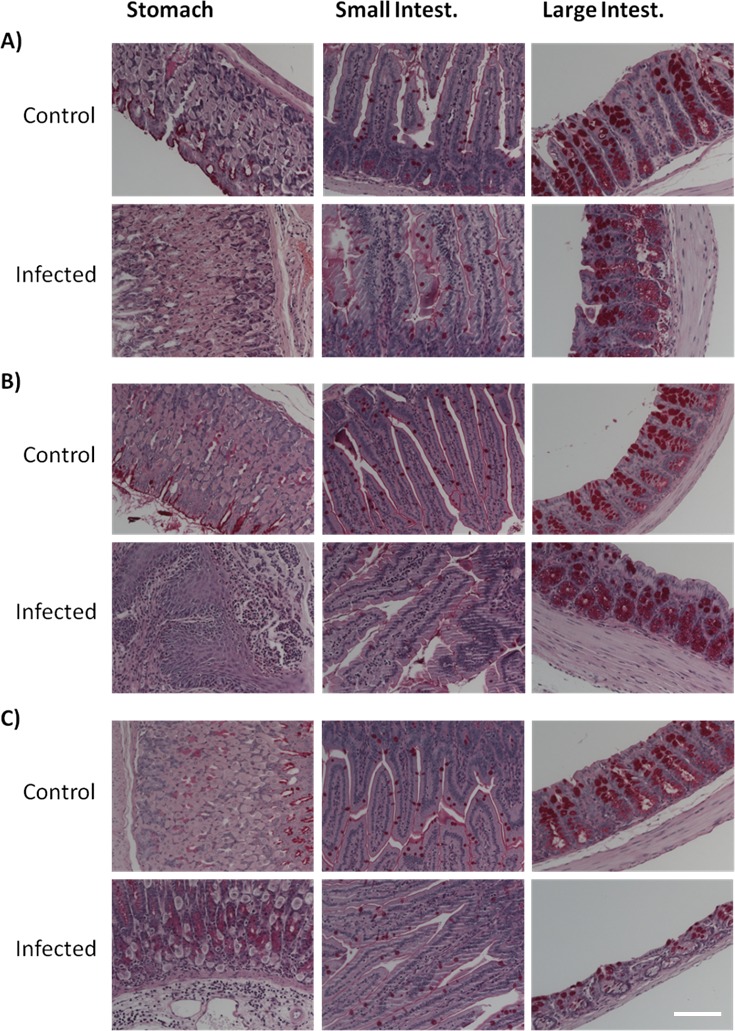
Histological analysis of periodic acid Schiff (PAS) stained tissues Stomach, small and large intestine sections from both control and *C. albicans* infected mice from **A.** aged, **B.** adult and **C.** young groups. No differences in glycogen and presence of mucopolysaccharides were observed between groups. Images were taken using a Zeiss Axio Imager Z2 Microscope; scale bar: 50 μm.

The analysis of the histological sections of the different GI tract tissues from control mice of the three age groups did not reveal any significant age-dependent alterations (Figures 3 and 4). According to the inflammatory criteria used to calculate a score index, we found a score of 0 (Table 1), with the absence of alterations in the tissues sections from control mice. By contrast, *Candida* infection induced a severe degree of disruption of the villi, haemorrhagic damage, and increase of inflammatory cells infiltration of the gastric mucosa (Figures 3; “Infected” panels). Infected aged animals had large inflammatory infiltrates (Figure 3A), and infected adult animals displayed the presence of lymphoid tissue (Figure 3B). In the small intestine, there was a mild disruption of villi and inflammatory infiltrate in the aged infected group, which did not reach significance compared to control mice (Figure 3 and Table 1). The large intestine from aged infected mice displayed mild epithelial cell loss and moderate haemorrhage (Figure 3 and Table 1). The alterations found in small and large intestines of infected mice from the different age groups revealed index scores of 0.5 to 2 (Table 1). When looking at stomach sections of young, adult and aged mice, the presence of *C. albicans* filamentous forms induced more severe alterations of villi, haemorrhage and inflammatory infiltrates (Table 1, score from 2 to 3), confirmed by the inflammatory processes illustrated in Figure 3. We did not observe differences between the groups with respect to the presence of glycogen or mucopolysaccharides in foveolar (stomach) and goblet cells (intestine) or presence of mucus when tissues were stained with PAS (Figure 4). Thus, both ageing and infection increased signs of inflammation in the GI tissues of the mice (Figures 3 and Table 1).

**Table 1:** Histological evaluation scores from mice gut tissues

		Control		Infected	
		Vilos.	Hem. Infilt.	Vilos.	Hem. Infilt.
**Stomach**	Aged	0 (0-0)	0 (0-0)	2	3
	Adult	0 (0-0)	0 (0-0)	3	3
	Young	0 (0-0)	0 (0-0)	3	3
**Small Intestine**	Aged	0 (0-0)	0 (0-0)	1 (0-1)	1.5 (1-2)
	Adult	0 (0-0)	0 (0-0)	0.5 (0-1)	1 (0-1)
	Young	0 (0-0)	0 (0-0)	0.5 (0-1)	0.5 (0-1)
**Large Intestine**	Aged	0 (0-0)	0 (0-0)	1 (0-1)	2 (0-2)*
	Adult	0 (0-0)	0 (0-0)	0 (0-1)	1 (1-2)
	Young	0 (0-0)	0 (0-0)	1 (0-1)	0 (0-0)^#^

Histological sections of stomachs, small and large intestines of control and infected mice groups (aged, adult and young) were analysed regarding villi (Vilos.), haemorrhage (Hem.) and inflammatory infiltrates (Infilt.).

Scores represent absence (0) to mild (1) and severe (3) alterations.

* *p* < 0.05 - adult infected *vs.* aged infected;

# *p* < 0.05 – aged infected *vs.* young infected.

### Mapping A_2A_ receptors (A_2A_R) in the gut of infected mice

**Figure 5:**
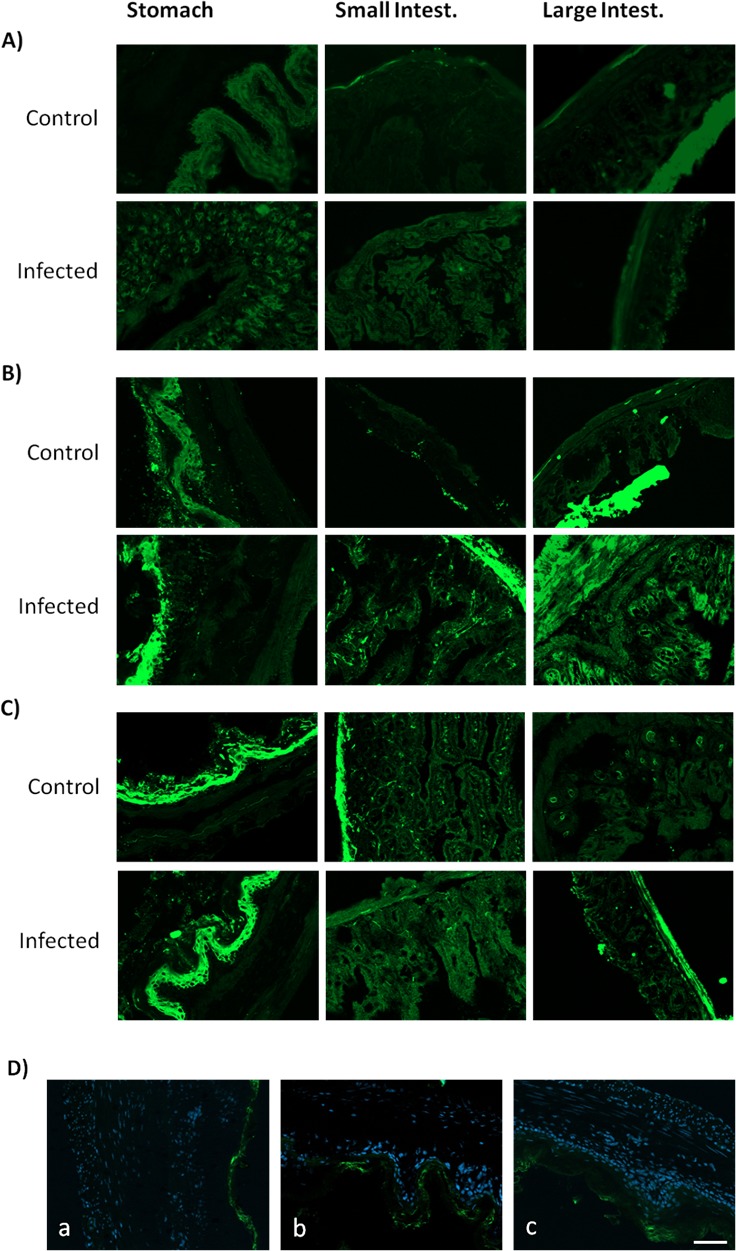
Adenosine A2A receptors localization on mice GI tract tissues Representative images of stomach, small and large intestine sections immunostained with an antibody against A_2A_R (green) of **A.** aged, **B.** adult and **C.** young mice. In adults and young mice, increased A_2A_R density during *C. albicans* infection was observed. In contrast, aged mice have lower levels and alterations of A_2A_R immunolabeling between conditions. **D.** Infected stomachs of a) aged, b) adult and c) young mice showing an increased A_2A_R density on epithelium and *muscularis mucosa* layers (cell nuclei stained blue) of young and adult compared to aged mice. Images were obtained using a Zeiss Axio Imager Z2 Microscope with ApoTome2 structured illumination acquisition system; scale bar: 10 μm.

Since infection situations trigger an up-regulation of A_2A_R, which feedback to curtail inflammation [10], we investigated if there was a correlation between A_2A_R density, inflammation, tissue damage and age. Adult and young animals displayed a larger A_2A_R immunoreactivity in GI tissues sections upon infection with *C. albicans* compared to controls (Figure 5B, 5C). This A_2A_R staining was particularly evident in the gastric epithelium layers of both adult and young mice, and also appeared with high intensity in the *muscularis mucosa* of stomach and small and large intestines from adult and young infected mice (Figure 5B, 5C, 5D). Notorious differences were observed when analyzing GI tissues from aged mice: Figure 5 shows that A_2A_R immunofluorescence was lower in GI tissues of aged mice compared to young and adult mice. Furthermore, the dynamic plasticity of A_2A_R density upon infection was lost in aged mice: *C. albicans* infection failed to increase A_2A_R staining in aged rats compared to non-infected aged rats (Figure 5A), in contrast to what was observed in young and adult mice (Figure 5B, 5C).

## DISCUSSION

This study tested the susceptibility to *C. albicans* over-growth or over-colonization of the gastrointestinal (GI) tract of mice with different ages. We aimed to correlate the over-colonization by *C. albicans* and subsequent inflammation in the GI tissues of young, adults and aged mice, with the modifications of adenosine A_2A_ receptors (A_2A_R), a major stop signal of inflammation. We used a well established and sustained murine GI infection model [14, 15, 16] based on the elimination of mice microbiota, which allowed an effective *C. albicans* GI colonization, without mice death owed to infection, in all age groups. *C. albicans* infection/over-colonization of elderly mice stomach lead to a more exuberant yeast-to-hypha transition with higher inflammation when compared to the other age groups. We also found that the overgrowth of *C. albicans* in the intestinal lumen of aged mice induced an inflammation of the mucosal tissues, which was independent on the invasion of intestinal tissues by yeast cells. The pattern of A_2A_R distribution in the GI tract showed that aged mice have a lower A_2A_R density in the GI tract tissues. Furthermore, A_2A_R density did not increase in response to *C. albicans* over-growth/infection, in contrast to the increased A_2A_R density observed in young and adult mice upon yeast infection.

The major conclusion of our study is that aged animals were more prone to yeast over-growth in the gut, which displayed a heighted inflammatory profile. First, we observed that *C. albicans* over-colonization of the gastric compartment (during the first 11 days of infection) in aged mice proceeds with a more exuberant morphological switch to the hyphal form, when compared with young and adult mice. Concomitantly, higher inflammation is observed in the gastric tissues of elderly mice. The importance of *C. albicans* morphologic switch for infection and dissemination in the gut is well recognized and has been lately highlighted [17, 18]. We observed a higher morphological switch and over-colonization of the stomach tissues than that observed in the intestinal segments, where the fungus was predominantly located in the intestinal lumen. This was attested by the elimination of high levels of viable *C. albicans* cells in the stools and low levels of intestinal tissue colonization. This novel aspect of the gut infection/colonization by *C. albicans* was also observed by Brown and co-workers [14, 15], and strongly suggests that the intestinal inflammation results from an overgrowth of yeast cells in the lumen rather than an invasion of the intestinal tissue. This was particularly evident in the aged mice.

It has been described that inflammation can facilitate translocation phenomena (*e.g*., [19, 20]), and that the colonization of the gut by yeasts is associated with translocation and subsequent hematogenous dissemination in immunosuppressed mice [13, 20]. In the present work we did not observe an invasive infection of the intestinal tissues by *C. albicans*. This prompts the conclusion that the inflammation of the intestine, especially in aged mice, can be triggered by the recognition of cell wall components of *C. albicans* or by extracellular secretion of products such as farnesol [21] or small peptides [22].

In contrast with previous findings regarding systemic *C. albicans* infection [22], we have found low yeast levels in the kidneys and livers of all animals, using an infection model *via* the drinking water. Those previous studies of invasive infections [22] report a substantial decrease of the colonization levels in kidneys and livers throughout the entire period of infection, with residual values over the end of the experiment. This suggests an efficient immunity response [22]. Since we only harvested the tissues at the end of the experiment, the low fungal burden quantified corresponds to a post-eradication period. Nevertheless, the low colonization rates that we now observed, were also previously reported by others [15, 23, 24].

The second major finding of our study is the tentative association of A_2A_R in GI tissues with the over-colonization by *C. albicans* and subsequent inflammation. The involvement of purines and their receptors in the pathophysiology of inflammatory gut diseases is best heralded by combined observations describing that, while extracellular ATP mediates inflammation [25], CD73, an ecto-nucleotidase responsible for converting AMP in adenosine, increases the recovery rate from severe colonic inflammation [26]. Adenosine is recognized as an anti-inflammatory agent, contributing to ameliorate the damage induced by inflammation [10, 27]. Accordingly, A_2A_R are involved in inhibitory mechanisms of pro-inflammatory cytokines secretion during colitis or enteritis, and the levels of adenosine in the intestinal luminal are increased in the inflamed intestine in several pathological conditions such as inflammatory bowel disease [28]. Our study provides the first qualitative analysis of A_2A_R distribution in the GI tract of mice from different age groups. We observed that aged mice had a lower A_2A_R density than young and adult mice. Moreover, instead of increasing the density of A_2A_R during *C. albicans* infection, as observed in adults and young mice, A_2A_R immunolabelling showed that A_2A_R density was maintained or slightly decreased. Therefore, we conclude that the inability to increase A_2A_R density in the GI tract of infected aged mice, contributes to a higher inflammatory score, with a lower ability to overcome the deleterious effects of *C. albicans* over-colonization, leading to a higher tissue damage. Overall, this supports the hypothesis that increased A_2A_R density at the surface of GI mucosa cells is a protective mechanism against *C. albicans* overgrowth; this mechanism is present throughout adulthood and decreases with age. It would be interesting to study the effect of drugs such as rapamycin, simultaneously displaying anti-aging [29] and anti-fungal [30] properties, over *C. albicans* colonization and A_2A_R distribution.

We also observed that in aged mice, yeast-to-hypha transition of *C. albicans* was more exuberant, especially in the stomach. We propose that this may result from the lower density of A_2A_R because adenosine and A_2A_R in the stomach contribute to decrease the secretion of gastric acid [31]. The lower A_2A_R density in the stomach of aged mice is expected to decrease the pH, leading to a stressful condition that triggers the morphological switch of *C. albicans*. In fact, it was described that, under acidic conditions, *C. albicans* raises the pH to neutral or alkaline, resulting in yeast-to-hypha transition, considered one of its most important virulent traits [32]. The age-related decrease of A_2A_R density observed by us, together with the ability of *C. albicans* to modulate the external pH [32], might explain our finding of a low rate of yeast-to-hypha transition in young mice, increasing in adults, and displaying a maximal rate in aged mice. This novel working hypothesis is of particular interest in the context of gastrointestinal diseases, which have increased incidence in elderly patients [33], especially in those with gastric ulcers who are more colonized by *C. albicans* [34].

In summary, the overgrowth of *C. albicans* in the intestinal lumen of aged mice induced inflammation of the mucosal tissues, which was independent on the invasion of intestinal tissues by yeast cells. The pattern of A_2A_R distribution in the GI tract showed that aged mice have less A_2A_R in the GI tract tissues, and that A_2A_R density does not increases in response to *C. albicans* overgrowth/infection, in contrast to the increased A_2A_R density observed in young and adult mice upon yeast infection. This paves the way to exploit the purinergic system, in particular the adenosine A_2A_ receptor, associated with beneficial effects under inflammatory conditions, to control over-colonization/infectious process of *C. albicans* in the gut of elderly. The impact of these findings is that the lack/reduced number of A_2A_R likely contributes to increased inflammation and lower ability to decrease *C. albicans* (or other) gut infection and the correspondent deleterious effects in the elderly.

## MATERIALS AND METHODS

### Strain, media and growth conditions

*C. albicans* YP0037 strain (Microbiology Pathogenic Yeast Collection, University of Coimbra) was isolated from a hemoculture. Yeast cells were grown overnight at 30°C on YPD agar (0.5% yeast extract, 1% bacto-peptone, 2% agar and 2% glucose) plates, harvested by centrifugation and resuspended in PBS (phosphate buffer saline, pH 7.4). Cell density was determined using a Neubauer chamber cell.

### Mice and gastrointestinal infection model

Young, adult and aged (respectively, 2-, 9- and 18-months-old) C57Bl/6JRj male mice were obtained from Janvier Labs (France) and maintained under specific pathogen-free conditions at the animal facilities of the Faculty of Medicine, University of Oporto. All procedures were according to EU guidelines (2010/63) and approved by the Directorate General of Food and Veterinary Medicine of the European Union (authorization no. 6411). Animals were individually housed in sterilized cages, supplied with sterile bedding, water and mouse chow and kept under a 12h:12h light-dark cycle, under controlled temperature (21-24°C) and humidity (50-60%). The gastrointestinal infection model was carried out as previously described [14] with slight modifications (Figure 1). Briefly, to reduce commensal bacterial and fungal flora, mice were treated for 3 days with sterile drinking water containing 2 mg/mL streptomycin (Reig Jofré, Spain), 2000 U/mL penicillin G (Atral, Portugal) and 0.25 mg/mL fluconazole (Pfizer, Germany) and then switched for a further day to water containing the same concentrations of streptomycin and penicillin G. Then, animals were provided, during 5 days, with sterile drinking water with *C. albicans* (1×10^7^ colony forming units, CFU/mL) and the same concentrations of streptomycin and penicillin G. After this period of yeasts ingestion, mice were maintained with sterile water containing 2 mg/mL streptomycin, 2000 U/mL penicillin G and 0.2 mg/mL gentamicin (Labesfal, Portugal) until the end of the experiment. Two additional groups of mice were maintained without *C. albicans* administration, one of the groups kept under the same antibiotic protocol while the other group was not exposed to antibiotics. To confirm the depletion of the endogenous microflora, stools were collected before beginning *C. albicans* administration.

To monitor *C. albicans* colonization the stools were collected from individual mice every two days. Stools were homogenized in 1 mL PBS and serial dilutions were made in order to obtain 30 to 50 CFU in each YPD agar plate. Plates were incubated for 2 days at 30°C and the yeast load was quantified by counting the number of colonies corresponding to viable yeast cells and expressed as CFU per g of stools. Mice were sacrificed 19 days after *C. albicans* exposure and kidneys, livers, stomachs, small intestines and large intestines were harvested and weighted. Equivalent sets of tissues were divided into three groups, (i) fixed in formalin solution for subsequent histology analysis; (ii) immediately snap-frozen in isopentane and kept at −80°C, for further immunohistochemistry; (iii) homogenized in PBS with 0.05% Triton X-100, diluted and plated on YPD as described above, to determine the tissue content of *C. albicans*.

### Histology

Formalized tissues were processed using standardized paraffin embedding protocols and stained with hematoxylin and eosin (HE) and periodic acid-Schiff (PAS) methods. Sections of the different organs were microscopically analyzed (Zeiss Axio Imager Z2 Microscope, with EC Plan-Neofluar, 5x/0.16 and 40x/0.75, objectives) in order to evaluate the presence of yeasts and/or inflammation. Stomach and small intestine sections were evaluated according to the criteria of Kelly [3]. Briefly, a 1-cm segment of each histological section was assessed on a scale of 0 (absence of alterations) and 1 (mild) to 3 (severe) for disruption of the villi, presence of red blood cells, and neutrophil infiltration. For large intestine, the presence and intensity of histological changes were evaluated with the use of a score index, according to Vilaseca [36], including the following criteria: presence of epithelial cell loss, presence and intensity of inflammatory infiltration and presence of blood cells.

### Immunohistochemistry

Snap-frozen samples were analysed using an immunohistochemistry standard protocol against A_2A_R. Briefly, OCT (Tissue-Tek, Sakura) ultrathin (10 μm) sections (Cryostat CM3050S, Leica Biosystem) were obtained from stomach, small and large intestine of the different mice. Tissue sections were fixed with pre-cooled acetone (−20°C) for 10 min, washed with PBS and permeabilized with PBS and 0.25% Triton X-100 for 30 min. After PBS washing, 10% albumin bovine serum (BSA) solution in PBS was used as blocking buffer and sections' slides were incubated in a humidified chamber at room temperature for 1 h. The rabbit anti-A_2A_R primary antibody (1:300; sc-13937 from Santa Cruz Biotechnology) was diluted in PBS with 1% BSA and incubated with the sections overnight at 4°C in a humidified chamber. Sections were then washed with PBS, and treated with the Alexa Fluor 488-labeled donkey anti-rabbit secondary antibody (1:2000; A21206 from Molecular Probes) diluted in PBS with 1% BSA. Sections were again incubated in a humidified chamber at room temperature for 2 h. After washing with PBS, nuclei were counterstained with DAPI (Sigma), during 5 min at room temperature, protected from light, and again rinsed with PBS. Coverslips were mounted with Vectashield HardSet mounting medium and kept at 4°C until visualized with fluorescence and/or confocal microscopy. Digital images were captured using a Zeiss Axio Imager Z2 Microscope (with ApoTome2 structured illumination acquisition system and a Plan-Apochromat 20x/0.8 objective), and using Zeiss ZEN 2012 (blue edition) and ImageJ software was used to analyse the images.

### Statistics

Data are means ± SEM. Statistical differences were determined with one or two-way ANOVA respectively followed by a Newman-Keuls or Bonferroni post hoc test. Histological damage scores were analysed with Kruskall Wallis and Mann-Whitney tests. Statistical significance was considered at *p* < 0.05.
